# Modelling competing risks in nephrology research: an example in peritoneal dialysis

**DOI:** 10.1186/1471-2369-14-110

**Published:** 2013-05-24

**Authors:** Laetitia Teixeira, Anabela Rodrigues, Maria J Carvalho, António Cabrita, Denisa Mendonça

**Affiliations:** 1 Doctoral Program in Applied Mathematics (PDMA), Institute of Biomedical Sciences Abel Salazar (ICBAS), University of Porto (UP), Porto, Portugal; 2 Institute of Public Health (ISPUP), University of Porto (UP), Porto, Portugal; 3 Research and Education Unit on Ageing (UNIFAI), Institute of Biomedical Sciences Abel Salazar (ICBAS), University of Porto (UP), Porto, Portugal; 4 Nephrology Unit, CHP – Hospital de Santo António, Porto, Portugal; 5 Unit for Multidisciplinary Investigation in Biomedicine (UMIB), Institute of Biomedical Sciences Abel Salazar (ICBAS), University of Porto (UP), Porto, Portugal; 6 Population Studies Department, Institute of Biomedical Sciences Abel Salazar (ICBAS), University of Porto (UP), Porto, Portugal

**Keywords:** Cause-specific hazard model, Competing risks, Cumulative incidence function, Peritoneal dialysis, Subdistribution hazard model, Survival analysis

## Abstract

**Background:**

Modelling competing risks is an essential issue in Nephrology Research. In peritoneal dialysis studies, sometimes inappropriate methods (i.e. Kaplan-Meier method) have been used to estimate probabilities for an event of interest in the presence of competing risks. In this situation a competing risk analysis should be preferable. The objectives of this study are to describe the bias resulting from the application of standard survival analysis to estimate peritonitis**-**free patient survival and to provide alternative statistical approaches taking competing risks into account.

**Methods:**

The sample comprises patients included in a university hospital peritoneal dialysis program between October 1985 and June 2011 (n = 449). Cumulative incidence function and competing risk regression models based on cause-specific and subdistribution hazards were discussed.

**Results:**

The probability of occurrence of the first peritonitis is wrongly overestimated using Kaplan-Meier method. The cause-specific hazard model showed that factors associated with shorter time to first peritonitis were age (≥55 years) and previous treatment (haemodialysis). Taking competing risks into account in the subdistribution hazard model, age remained significant while gender (female) but not previous treatment was identified as a factor associated with a higher probability of first peritonitis event.

**Conclusions:**

In the presence of competing risks outcomes, Kaplan-Meier estimates are biased as they overestimated the probability of the occurrence of an event of interest. Methods which take competing risks into account provide unbiased estimates of cumulative incidence for each specific outcome experienced by patients. Multivariable regression models such as those based on cause-specific hazard and on subdistribution hazard should be used in this competing risk setting.

## Background

Survival analysis is a statistical method widely used in medical literature that explores the time period from a certain point until the occurrence of the event of interest [1,2].

In peritoneal dialysis (PD) programs, for example, such approach is used to evaluate patient and technique survival, peritonitis-free survival and hospitalization-free survival [3-10].

In various areas of Nephrology research, we are in the presence of multiple competing events. A competing risk is an event whose occurrence either precludes the occurrence of another event under examination or fundamentally alters the probability of occurrence of this other event [11]. For example, analyzing patient survival in PD program, the event of interest is death in PD but other events can be observed: renal transplantation or transfer to haemodialysis. If one of these two events occurs, the event of interest cannot be observed.

In PD, few published studies address the competing risks approach, with emphasis on the research published by Evans et al. [12]. A very recent publication by Beuscart et al. [13] also addresses this issue, however this paper only focuses on univariable methods for survival analysis. The current paper discusses multivariable methods and provides further insights in PD survival analysis taking competing risks into account.

As peritonitis is a major complication in PD program, it is mandatory to adequately control peritonitis rate and to evaluate peritonitis-free survival [14].

The objectives of this study are to describe the bias resulting from the application of standard survival analysis (Kaplan Meier method) to estimate peritonitis**-**free patient survival in a PD program and to provide alternative statistical approaches taking competing risks into account. Regression models based on cause-specific hazard and subdistribution hazard were performed and the estimates obtained by such models were examined and discussed.

## Methods

The sample comprised all patients who started PD between October 1985 and June 2011 in Peritoneal Dialysis Unit, Nephrology Department, CHP – Santo António Hospital, Porto, Portugal. Consecutive incident end-stage renal disease (ESRD) patients starting PD were identified from an ongoing registry based prospective study of quality assessment. Each consecutive patient admitted in this unit is systematically enrolled for the purpose of the program control, with regular monthly input of data related with peritonitis events, hospitalizations, catheter complications, death, renal transplantation or transfer of modality with respective causes. Patients follow 1-2 monthly regular visits and clinical pathways according to the International Guidelines are used [15]. Within the quality control procedures, the profile of clinical complications and survival curves are audited. Patient outcome was defined as the earliest event among: first episode of peritonitis, death, transfer to haemodialysis and renal transplantation. Peritonitis was defined based on International Guidelines [15]. Transfer to haemodialysis was defined as definite drop-out from peritoneal dialysis. Registry data collection and analysis was submitted to ethical appreciation and approved by the National Commission of Data Protection, which is the national supervisory authority for personal data control.

### Statistical methods

#### Competing risks and cumulative incidence function

Standard survival analysis methods have been commonly used to analyze competing risks data. But, sometimes, inappropriate methods such as the complement of Kaplan-Meier estimate (1-KM) have been applied to estimate probabilities of the occurrence of an event of interest in a competing risks setting [11]. The 1-KM cannot be interpreted as the actual probability of the occurrence of an event by time *t*[16]. In this classical analysis there is an event of interest and all other events are censored. The assumption of this method is of non-informative censoring. This assumption considers that censored patients have the same probability of experiencing the event as patients who remain under follow-up. However, in the presence of multiple competing outcomes, this assumption is not verified [17]. These estimates have been interpreted as the probability of an event of interest in an ideal world where the other types of events do not exist [16,18]. Although, in the presence of competing risks, each event has a hazard. Therefore, the number of failures from the competing risks will reduce the actual number of failures from the event of interest and consequently, influence the estimate of the probability of failure from this event [11]. In these situations, the cumulative incidence function is the appropriate tool to analyse such data. Cumulative incidence function for a specific event, also known as the subdistribution function [16], is defined as the probability of failing from a given cause in the presence of competing events, given that a subject has survived or has already failed due to different causes [16,19,20].

In the present study, the estimate of the cumulative incidence for a specific event was calculated based simultaneously on the estimate of the overall survival function when all types of events are considered and on the hazard estimate of the specific event [16,21]. Then, the cumulative incidence function for a specific event depends not only on the number of individuals who have experienced this type of event, but also on the number of individuals who have not experienced any other event [16].

This function is often of interest in medical research and its graphical display over time is intuitive and appealing [20,22].

To analyze differences in the cumulative incidence between various patient groups, Gray’s test was used [19]. Comparing the cumulative incidence functions gives an idea of the probability of occurrence of the event of interest and therefore can be translated into an actual number of patients with the event of interest [16].

#### Multivariable regression models

In the multivariable analysis, two types of models were performed considering two types of hazard: cause-specific hazard and hazard of the subdistribution. The cause-specific hazard at time *t* is a fundamental concept in competing risks, defined by the instantaneous risk of failure per time unit from the event of interest given survival till just before *t*[23]. This hazard measures the instantaneous failure rate due to one risk [1]. The hazard of the subdistribution is interpreted as the probability of observing an event of interest in the next time interval while knowing that either the event of interest did not happen until then or that a competing risks event was observed [16].

##### Models on cause-specific hazards

The standard analysis for competing risks data involves modelling the cause-specific hazard functions of the different failure types [24]. Proportional cause-specific hazards regression models can be estimated using the standard Cox regression and censoring patients with competing events at the time point of their occurrence [25]. The utilization of Cox regression models for cause-specific hazards has the advantage that they are easy to fit and they provide parameter estimates which possess simple rate ratio interpretations. Such models, however, do not provide simple relationships between covariates and the easier interpretable cumulative incidences [23].

##### Models on subdistribution hazards

The model proposed by Fine and Gray [24] is based on the hazard of the subdistribution and provides a simple relationship between covariates and cumulative incidence [23]. As in any other regression analysis, modelling cumulative incidence functions for competing risks can be used to identify potential prognostic factors for a particular failure in the presence of competing risks, or to assess a prognostic factor of interest after adjusting for other potential risk factors in the model [20]. However, this analysis cannot be fully interpreted without examining the effect of the covariates on the competing risks [16,26].

In the present study, to analyze peritonitis-free survival, the event of interest was the first episode of peritonitis and the competing risk events were death, transfer to haemodialysis and renal transplantation. Patients with partial recovery of renal function before the first episode of peritonitis were excluded (given the small number involved (n = 6)). Patients without any of these outcomes were censored at the date of their last recorded visit or at the end of the study period (June 2011).

Other variables, such as gender, age groups (<55 years; ≥55 years) [27], diabetes (yes; no), and first renal replacement therapy (PD; haemodialysis; renal transplantation), were evaluated.

Survival analysis methods taking competing risks into account were performed for analyzing peritonitis-free survival. First, estimates of cumulative incidence function were calculated and compared with 1-KM estimates. Then, subgroup analyses, using Gray’s test, were conducted considering the patient characteristics. Finally, regression models taking competing risks into account (Cox cause-specific hazard model and Fine and Gray model based on subdistribution hazard model) were carried out to analyze the effect of covariates in the peritonitis-free survival. To decide which variables should be included in the multivariable models, an exploratory analysis was performed by fitting univariable models and considering as candidates for the multivariable model all variables significant at the 0.10 significance level in these univariable models. For the final multivariable models, the significance level was set at 0.05 and they were built including all variables with p-values <0.05.

All analyses were performed with R software using the packages *coxph* and *cmprsk*.

## Results

### Sample

The final sample comprises 449 patients, 61.0% women (n = 274) and the mean age was 48.2 years (sd = 15.8 years). Median follow-up was 10 months (range 0-118 months). First peritonitis episode was the commonest outcome (n = 238, 53.0%). Renal transplantation was the main reason for PD discontinuation (n = 65, 14.5%), followed by transfer to haemodialysis (n = 58, 12.9%) and death (n = 46, 10.2%). At the end of the study period, 9.4% of the patients were still on PD having not experienced a peritonitis episode (n = 38) or were lost to follow-up (n = 4). More than half of the patients were PD first (i.e. the first renal replacement therapy was PD), 22.5% had diabetes and 57.9% had started PD by option (Table 1).

**Table 1:** Characteristics of the patients

	**n**	**%**
Gender		
Male	175	39.0
Female	274	61.0
Age*	48.2	(15.8)
Outcome		
First peritonitis	238	53.0
Death	46	10.2
Transfer to haemodialysis	58	12.9
Renal transplantation	65	14.5
Censored	42	9.4
Diabetes		
Yes	100	22.5
No	345	77.5
First treatment		
Peritoneal dialysis	244	54.4
Haemodialysis	151	33.6
Renal transplantation	54	12.0
Reason for peritoneal dialysis		
Option	259	57.9
Access failure	188	42.1

*Mean (SD).

### Cumulative incidence estimates

**Figure 1:**
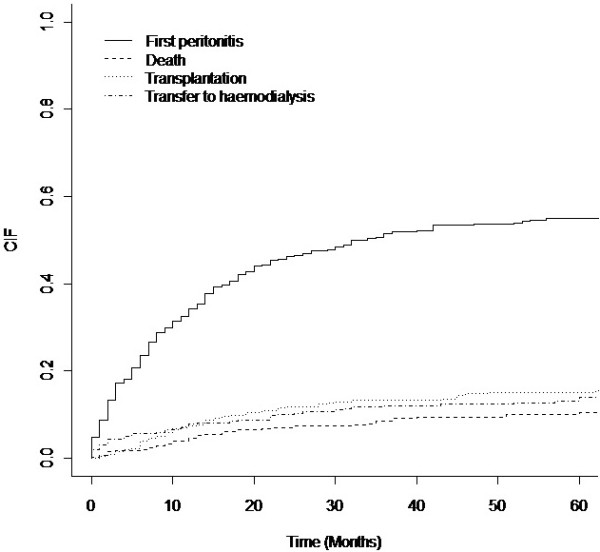
Cumulative incidence curves for all possible outcomes, taking competing risks into account.

Figure 1 summarizes the cumulative incidence estimates for all possible outcomes taking competing risks into accounts (the survival plots were halted at 60 months because the proportion of patients free of an event, but still in follow-up, becomes small). For example, the probabilities of experiencing peritonitis by 1, 3 and 5 years after starting PD were 0.34, 0.52 and 0.55, respectively.

### Kaplan-Meier method *vs* cumulative incidence function

**Figure 2:**
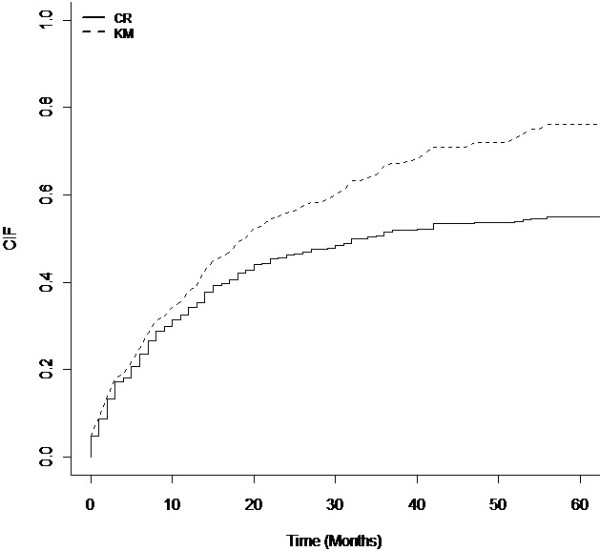
The complement of the Kaplan-Meier estimate and the cumulative incidence estimate for first peritonitis.

Figure 2 presents the curves for the cumulative incidence function of the occurrence of the event of interest obtained using two different methods: method taking competing risks into account and the complement of Kaplan-Meier method.

The appropriate competing risks approach results in a lower estimate of cumulative incidence. The magnitude of the differences between incidences of first peritonitis as calculated using the two methods increases with period of follow-up. In other words, the actual probability of occurrence of the first peritonitis is wrongly overestimated using Kaplan-Meier method and the longer the duration of follow-up the larger the difference between the estimated by these two methods.

Subgroup analyses, using Gray’s test, were performed calculating cumulative incidence for all possible events according to the variables gender, age, diabetes, first treatment and reason for PD.

**Figure 3:**
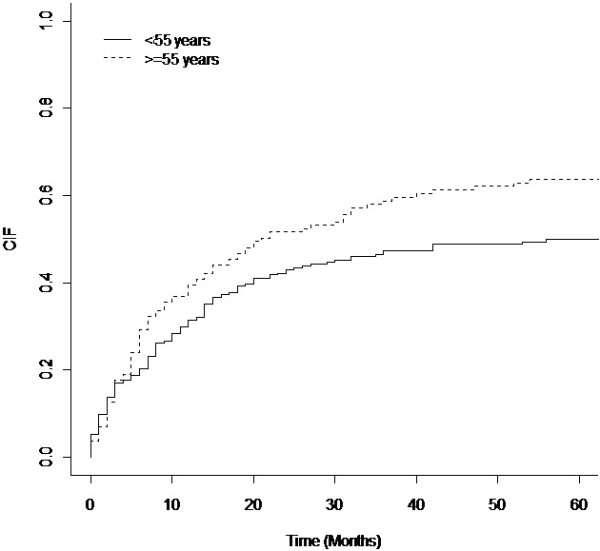
Cumulative incidence curves for first peritonitis outcomes, according to age groups.

Considering first peritonitis as the event of interest only age groups was statistically significant (p = 0.008). Figure 3 shows the cumulative incidence curves for the first peritonitis in the two age groups and it can be seen that the group of older patients (≥55 years) presents always a higher risk of peritonitis.

When considering the competing risks events, death and renal transplantation, age was a significant factor conditioning the probability of these events (p = 0.005 and p < 0.001, respectively). Older patients showed a higher hazard risk of death and a lower hazard risk of renal transplantation compared with younger patients. Patients with diabetes presented a higher hazard risk of death (p = 0.037).

### Regression models

Tables 2 and 3 give a summary of the unadjusted and adjusted effects of covariates for first peritonitis based on the two types of modelling: cause-specific hazard models and subdistribution hazard models.

**Table 2:** Cox proportional hazard regression (cause-specific hazard model) for the event of interest (first peritonitis)

		**Unadjusted model**			**Adjusted model**		
		**HR**	**95% CI**	**p**	**HR**	**95% CI**	**p**
Gender	Female	1.24	0.95-1.62	0.12			
	Male	1	-	-			
Age	≥55 years	1.37	1.06-1.78	0.017	1.35	1.03-1.76	0.030
	<55 years	1	-	-	1	-	-
Diabetes	Yes	0.92	0.66-1.28	0.63			
	No	1	-	-			
First treatment	HD	1.45	1.10-1.91	0.009	1.44	1.09-1.90	0.009
	TR	0.95	0.62-1.46	0.82	1.06	0.68-1.64	0.791
	PD	1	-	-	1	-	-
Reason for PD	Access failure	1.21	0.93-1.57	0.15			
	Option	1	-	-			

**Table 3:** Fine and Gray model (hazard of the subdistribution model) for all possible events

			**Unadjusted model**			**Adjusted model**		
			**sHR**	**95% CI**	**p**	**sHR**	**95% CI**	**p**
First peritonitis	Gender	Female	1.26	0.97-1.63	0.085	1.32	1.02-1.72	0.037
		Male	1	-	-	1	-	-
	Age	≥55 years	1.41	1.10-1.82	0.007	1.47	1.14-1.89	0.003
		<55 years	1	-	-	1	-	-
	Diabetes	Yes	0.79	0.57-1.09	0.15			
		No	1	-	-			
	First treatment	HD	1.28	0.98-1.68	0.069			
		TR	0.99	0.65-1.53	0.98			
		PD	1	-	-			
	Reason for PD	Access failure	1.13	0.87-1.45	0.36			
		Option	1	-	-			
Death	Gender	Female	1.08	0.58-1.99	0.81	1.19	0.64-2.23	0.58
		Male	1	-	-	1	-	-
	Age	≥55 years	2.23	1.23-4.02	0.008	2.27	1.24-4.15	0.007
		<55 years	1	-	-	1	-	-
	Diabetes	Yes	1.85	1.00-3.42	0.05			
		No	1	-	-			
	First treatment	HD	1.47	0.79-2.72	0.22			
		TR	0.84	0.29-2.45	0.75			
		PD	1	-	-			
	Reason for PD	Access failure	1.81	1.00-3.29	0.05			
		Option	1	-	-			
Renal transplantation	Gender	Female	0.92	0.56-1.50	0.73	0.82	0.50-1.33	0.42
		Male	1	-	-	1	-	-
	Age	≥55 years	0.19	0.09-0.42	<0.001	0.19	0.09-0.41	<0.001
		<55 years	1	-	-	1	-	-
	Diabetes	Yes	0.79	0.42-1.49	0.46			
		No	1	-	-			
	First treatment	HD	0.58	0.32-1.06	0.076			
		TR	1.08	0.56-2.11	0.82			
		PD	1	-	-			
	Reason for PD	Access failure	0.65	0.39-1.09	0.10			
		Option	1	-	-			
Transfer for haemodiaysis	Gender	Female	0.81	0.48-1.36	0.43	0.79	0.47-1.32	0.36
		Male	1	-	-	1	-	-
	Age	≥55 years	0.73	0.41-1.28	0.27	0.71	0.41-1.24	0.23
		<55 years	1	-	-	1	-	-
	Diabetes	Yes	1.64	0.94-2.85	0.08			
		No	1	-	-			
	First treatment	HD	1.17	0.67-2.04	0.59			
		TR	1.23	0.57-2.65	0.90			
		PD	1	-	-			
	Reason for PD	Access failure	1.28	0.76-2.14	0.35			
		Option	1	-	-			

Considering the unadjusted models (univariable) for the event of interest (first peritonitis), it was found that the variables age and first treatment were significant in the cause specific-hazard model (Table 2) and only the variable age was significant in the subdistribution hazard model (Table 3).

The results show that the effects of age for the cause-specific and subdistribution hazard models are quite close for the event of interest, first peritonitis.

Considering the cause-specific multivariable model, risk of peritonitis increased with age (HR = 1.35, 95% CI = 1.03-1.76). Risk of peritonitis was also higher for patient with haemodialysis as first treatment compared with PD first (HR = 1.44, 95% CI = 1.09-1.90) (Table 2).The effect of gender when adjusted for age groups and first treatment remains non-significant (HR = 1.28, 95% CI = 0.97-1.69).

Unlike the cause-specific hazard model, the subdistribution multivariable model found both gender and age group statistically significant but not first treatment. Females were at 32% higher hazard risks of first peritonitis compared to male (sHR = 1.32, 95% CI = 1.02-1.72) and age equal or greater than 55 had 1.47 times higher risk of peritonitis (sHR = 1.47, 95% CI = 1.14-1.89) compared to those younger than 55 years (Table 3).

This analysis cannot be fully interpreted without examining the effect of these covariates on the competing risks because the results are influenced by the way the competing risks were distributed. Given that women have a slightly lower probability of experiencing the competing risks (renal transplantation and transfer to haemodialysis), the event of interest could be observed and therefore the effect of gender becomes larger. Analyzing the competing events, patients aged equal or greater than 55 years had lower risk of renal transplantation compared to those aged younger than 55 in the subdistribution hazard model (sHR = 0.19, 95% CI = 0.09-0.41). All the remaining variables were found not to be statistically significant.

### Discussion and conclusions

This study addresses a clinically relevant methodological issue, focused on peritonitis events under PD. While peritonitis has been chosen as an example of a main event, our study discusses the application of competing risk analysis in dialysis populations by exploring multivariable models that allow clinicians to answer relevant questions related with any other serious event: who is at a higher risk of peritonitis, death, transfer to HD? But if a certain patient is younger and will have faster access to transplantation, for example, will that risk be the same? Dialysis policies and patients certainly need this kind of approaches to more accurately predict the risks.

When analysing peritonitis-free patient survival, a relevant overestimation was presented when Kaplan-Meier method was used, revealing the importance of using the competing risks approach in survival analysis in PD. In the presence of competing risks outcomes, the Kaplan-Meier core assumption of non-informative censoring does not hold: the presence of competing risk events results in informative censoring [1,28]. Previous work has shown that 1-KM and cumulative incidence function, each of which is commonly used to estimate the probability of failure, can result in different estimates when competing risks are present. The bias resultant of this approach is especially great when the hazard of the competing risks is large [26].

In our study and using the more appropriate methodology we were able to document that the probabilities of experiencing a first peritonitis by 1, 3 and 5 years after starting PD were 0.34, 0.52 and 0.55, respectively.

Additionally age (≥55 years) and gender (female) were identified as factors associated with a higher probability of first peritonitis occurrence, but not diabetes or previous renal replacement modality in the subdistribution multivariable regression model. This methodology indicates that in our program the use of PD was feasible in diabetics and non-naïve PD patients, coming from haemodialysis or after renal graft failure, without significantly higher risk of experiencing a first peritonitis episode. Previous studies have identified association between peritonitis and other risk factors [12], but this study was mainly designed to perform a critical appraisal of the methodology used in the competing risk setting and not to fully examine all independent risk factors for peritonitis.

As we have mentioned, two approaches can be used when competing risks are present: modelling the cause-specific hazard and modelling the hazard of the subdistribution (taking into account the competing risks). But the covariate effects using the cause-specific hazards or the subdistribution hazards models may be different [26], as found in this study. The objective of the first approach is to test whether the peritonitis incidence rate is different between groups. So, we may point to an etiologic factor or relevant clinical factor with biological relevance. In the second approach the interest is to estimate how many more (or less) peritonitis cases are seen in each group. The presence of competing risks influences the number of events observed, since a person who has experienced a competing risk (death, transfer to haemodialysis or renal transplantation) will no longer have the chance of having peritonitis. The results obtained in the subdistribution hazard model are influenced by the way the competing risks were distributed. If patients with a characteristic were more likely to have a competing risk, the event of interest could not be observed and therefore the effect of this covariate would be diminished [16]. This may explain why the variable first treatment was found to be statistically significant in the cause-specific hazard model but not in the subdistribution model.

Both models were performed because the choice between the two approaches is based on the research question and the two analyses may complement each other. For etiologic questions, a cause-specific hazards model is generally more appropriate, since it quantifies the event rate among the ones at risk of developing the event of interest. However since the focus is often on the direct assessment of actual risks and therefore the purpose of prediction and medical decision making, subdistribution regression models for the cumulative incidence function are preferred [23,25,29].

In fact, cause-specific hazards (CSH) and cumulative incidence functions (CIF) provides different aspects of the event histories in competing risks problems and inference on these measures may yield different conclusions. Given the complementary nature of these approaches, a universal recommendation for all problems would not be appropriate and inference should be based on a priori choice of the primary question to be addressed.

In competing risks problems, CSH is a fundamental measure and the most commonly used. It is appropriate for investigating the effect of a covariate on the rate of occurrence of an event in the presence of all types of events. CIF is useful for evaluating the effect of a covariate on the probability of the occurrence of an event of interest over a meaningful period of time. This is the best measure for absolute risk calculations and risk prediction. Results of CIF analysis should always be reported for all events (interest and competing events) [19,30,31].

In the area of PD, there are few published works that address the competing risks approach. In their study, Evans et al. [12] analyzed the peritonitis-free survival and the results obtained are similar to our study, revealing the overestimation of cumulative incidence estimation obtained by Kaplan-Meier method. Quinn et al. [32] present a reflection about the importance and relevance of survival analysis taking competing risks into account, when competing risks events are present, illustrating this situation with an example on PD. Beuscart et al. [13] also discussed competing risk approach and applied univariable survival analysis. In our study we discuss further this topic and illustrated how the methodology should be used when investigating other serious events in dialysis populations using a multivariable approach.

The relevance of the present critical investigation is that it can be applied to any other PD serious event such as death or technique failure. Furthermore, the multivariable approach presented in the current paper is relevant to other areas of nephrology namely in the evaluation of haemodialysis programs (where renal transplantation and transfer to PD are competing risks) or in renal transplantation when analysing graft failure (where death with functioning graft could be considered a competing event).

Apart from these clinical issues we highlight that methods which take competing risks into account provide unbiased estimates of cumulative incidence for each specific outcome experienced by patients. With competing risks packages available in some standard statistical software (e.g. SPSS, SAS, STATA or R), it is hoped that they may become more widely used in renal research.

In conclusion, a competing risk approach to estimating cumulative incidence in studies with multiple outcomes, specifically in PD studies, will result in more rigorous estimates and is recommended.

## Competing interests

The authors declare that they have no competing interests.

## Authors’ contributions

LT was involved in the design of the study, performed the statistical analysis and drafted the manuscript. DM was involved in the discussion of statistical analyses contents and in the revision of the manuscript for intellectual contents. AR, MJC and AC were involved in the revision of the manuscript for intellectual contents and in the recruitment of patients and collection of clinical data. All authors approved the final manuscript.

## Pre-publication history

The pre-publication history for this paper can be accessed here:

http://www.biomedcentral.com/1471-2369/14/110/prepub
